# Simvastatin Inhibits Cell Proliferation and Migration in Human Anaplastic Thyroid Cancer

**DOI:** 10.3390/ijms18122690

**Published:** 2017-12-13

**Authors:** Mei-Chieh Chen, Yuan-Chin Tsai, Jen-Ho Tseng, Jr-Jiun Liou, Steve Horng, Heng-Ching Wen, Yu-Ching Fan, Wen-Bin Zhong, Sung-Po Hsu

**Affiliations:** 1 Department of Microbiology and Immunology, School of Medicine, College of Medicine, Taipei Medical University, Taipei 110, Taiwan; mcchen@tmu.edu.tw; 2 Graduate Institute of Medical Sciences, College of Medicine, Taipei Medical University, Taipei 110, Taiwan; b409097033@tmu.edu.tw (J.-J.L.); d119099014@tmu.edu.tw (H.-C.W.); 3 Graduate Institute of Cancer Biology and Drug Discovery, College of Medical Science and Technology, Taipei Medical University, Taipei 110, Taiwan; yuanchin@tmu.edu.tw (Y.-C.T.); steve4211@tmu.edu.tw (S.H.); ssfan1616@gmail.com (Y.-C.F.); 4 Department of Neurosurgery, Taipei City Hospital, Renai Branch, Taipei 106, Taiwan; DAL87@tpech.gov.tw; 5 Department of Physiology, School of Medicine, College of Medicine, Taipei Medical University, Taipei 110, Taiwan

**Keywords:** simvastatin, RhoA, p21^cip^, p27^kip^, anaplastic thyroid cancer

## Abstract

Malignant human anaplastic thyroid cancer (ATC) is pertinacious to conventional therapies. The present study investigated the anti-cancer activity of simvastatin and its underlying regulatory mechanism in cultured ATC cells. Simvastatin (0–20 μM) concentration-dependently reduced cell viability and relative colony formation. Depletions of mevalonate (MEV) and geranylgeranyl pyrophosphate (GGpp) by simvastatin induced G1 arrest and increased apoptotic cell populations at the sub-G1 phase. Adding MEV and GGpp prevented the simvastatin-inhibited cell proliferation. Immunoblotting analysis illustrated that simvastatin diminished the activation of RhoA and Rac1 protein, and this effect was prevented by pre-treatment with MEV and GGpp. Simvastatin increased the levels of p21^cip^ and p27^kip^ proteins and reduced the levels of hyperphosphorylated-Rb, E2F1 and CCND1 proteins. Adding GGpp abolished the simvastatin-increased levels of p27^kip^ protein, and the GGpp-caused effect was abolished by Skp2 inhibition. Introduction of Cyr61 siRNA into ATC cells prevented the epidermal growth factor (EGF)-enhanced cell migration. The EGF-induced increases of Cyr61 protein expression and cell migration were prevented by simvastatin. Taken together, these results suggest that simvastatin induced ATC proliferation inhibition through the deactivation of RhoA/Rac1 protein and overexpression of p21^cip^ and p27^kip^, and migration inhibition through the abrogation of Cyr61 protein expression.

## 1. Introduction

Human anaplastic thyroid cancer (ATC) is a highly aggressive and malignant disease. The survival time of patients since clinical diagnosis of the disease is 2–6 months only [1,2]. Patients with ATC frequently accompany the metastases in lung, bone and brain. The death of ATC patients is mostly a result of airway hindrance or complications caused by the distant metastases of ATC [3]. Conventional managements for ATC patients are surgery, chemotherapy, radiotherapy and a combination of these regimens [4]. Although neck dissection or thyroidectomy could be possible in quite a few populations among patients with intrathyroidal ATC, a complete resection of ATC is less likely because of a direct embroilment of crucial structures, esophagus and carotid arteries [5]. Radiotherapy may minimize the cell populations of ATC, but cannot restrain it [6], and the resulting toxicity is also difficult to be estimated [7]. The consequent resistance to chemotherapy drugs, such as doxorubicin, disappoints the possible response rates [8]. Chemotherapy in combination with radiotherapy may ameliorate the adverse effects of radiotherapy in loco-regional and distant control, but the effects of the combined therapies on survival rate are dismal [9]. Novel strategies, such as targeted therapies, have been developed, but the clinical trials are required to be further conducted, and it will take more years [4]. Progression of treatment strategies to fight ATC is limited and relatively slow. However, even an atom of progression would still encourage patients to vanquish ATC.

Statins, a group of 3-hydroxy-3-methylglutaryl (HMG)-CoA reductase inhibitors, are clinically used for hypercholesterolemia reduction, decreasing the associated mortality of patients with cardiovascular diseases [10]. Simvastatin binds to the functional site of HMG-CoA reductase, subsequently inducing a conformational change, and then hinders the binding of HMG-CoA to HMG-CoA reductase which precludes the conversion of mevalonate (MEV), and finally removes excess circulating cholesterol. The interference of MEV production results in deprivation of its non-sterol and downstream isoprenoid intermediates, such as farnesyl pyrophosphate (Fpp) and geranylgeranyl pyrophosphate (GGpp) [11]. These two MEV-derived metabolites, Fpp and GGpp, are critical substrates for protein isoprenylation (farnesylation and geranylgeranylation), which plays important roles in the regulation of diverse cellular progressions such as membrane targeting, protein–protein interaction, cytoskeleton remodeling and cell growth [12,13]. Deregulation or defects in protein isoprenylation might lead to the development of neurodegenerative diseases [14,15], metabolic diseases [16,17] and cancers [18,19]. To meet the survival and growth demands, cancer cells alter their metabolic pathway to gain oncometabolites for required energy production and nutrient biosynthesis [20]. The converting enzymes, which catalyze the synthesis of MEV-derived oncometabolites, have been lately highlighted in the survival requirements of cancer cells [21,22]. Moreover, observations from preclinical studies and clinical meta-analysis showed that the potent activity of statins, which inhibit the flux-initiating enzyme HMG-CoA reductase of the MEV pathway, could suppress the survival of cancer cells by inducing growth arrest and apoptosis [19,23]. These findings suggest that the MEV pathway is integral and drug-targetable for cancer therapy. 

In the present study, we investigated the effects of simvastatin on fast-growing and malignant ATC cell lines and identified key molecules of the MEV pathway involved in the simvastatin-induced inhibition of ATC cell proliferation and migration in vitro. Our results suggest that simvastatin reduced ATC cell proliferation via up-regulations of p21^cip^ and p27^kip^ expression, and migration via down-regulation of Cyr61 expression.

## 2. Results

### 2.1. Inhibition of ATC Cell Growth by Simvastatin

**Figure 1:**
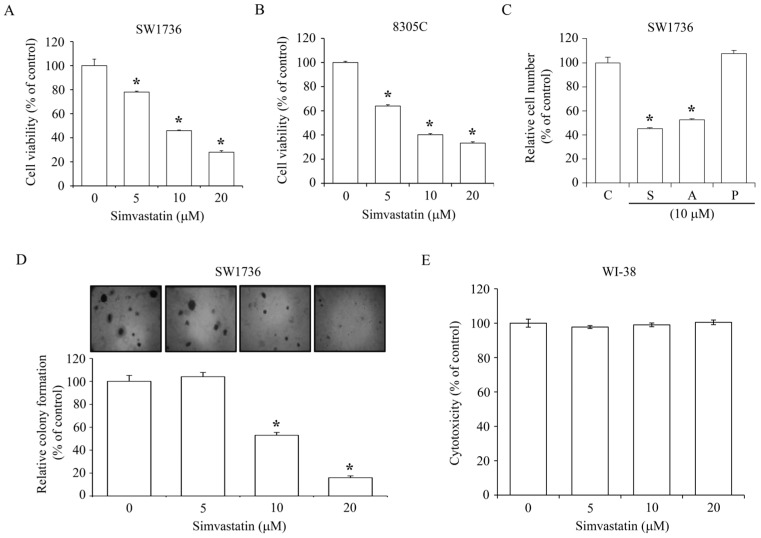
Effects of simvastatin on ATC cell viability and colony formation. Simvastatin concentration-dependently reduced the viability of SW1736 (**A**) and 8305C (**B**) cells. Cells were incubated with 0–20 μM simvastatin for 48 h, and then the cell viability was accessed by MTT assay. (**C**) Simvastatin (S) and atorvastatin (A) reduced the cell number of SW1736 cells, whereas pravastatin (P) had no effect on the cell number. Cells were incubated with drug compounds for 48 h, and then the relative cell number was accessed by MTT assay. (**D**) Simvastatin reduced the growth of SW1736 cells in the semi-solid agar. Cells were incubated with 0–20 μM simvastatin for 14 days, and then the cell colonies were stained using crystal violet. (**E**) Simvastatin did not cause the cytotoxic effect. SW1736 cells were incubated with 0–20 μM simvastatin for 24 h, and then the cytotoxicity was accessed by MTT assay. Values represent the means ± standard error of the mean (SEM) (*n* = 3). * *p* < 0.05, different from corresponding control.

To observe the effect of simvastatin on ATC cell growth, SW1736 and 8305C lines were incubated with simvastatin at various concentrations (0–20 μM). 3-(4,5-Dimethylthiazol-2-yl)-2,5-diphenyltetrazolium bromide (MTT) assays showed that simvastatin concentration-dependently reduced the growth rate of SW1736 and 8305C cells. At a concentration of 20 μM, simvastatin-induced growth inhibition of SW1736 and 8305C was 70% and 65%, respectively (Figure 1A,B). We further observed the effect of atorvastatin and pravastatin on the inhibition of SW1736 cell number at 48 h. As illustrated in Figure 1C, both simvastatin and atorvastatin could cause a decrease of the cell number up to 50% compared to the control group, whereas pravastatin had no significant effect on the number of SW1736 cells. To investigate the effect of simvastatin on ATC anchorage-independent growth, which is fundamentally considered as a symbol of cell transformation and metastasis development in vivo [24], colony formation in semi-solid agar was assayed. As shown in Figure 1D, simvastatin at a concentration of 5 μM did not produce significant inhibition of cologenic growth in SW1736; however, simvastatin at 10 and 20 μM significantly reduced the colony formation up to 50–80% as compared to the vehicle-treated control group. These results suggest that simvastatin might also inhibit metastatic transition. Treatment of WI-38 normal human lung fibroblast cells with simvastatin at various concentrations (0–20 μM) did not cause a cytotoxic effect at 24 h (Figure 1E). 

### 2.2. Effects of MEV and Its Metabolites on the Simvastatin-Induced ATC Cell Proliferation Inhibition

**Figure 2:**
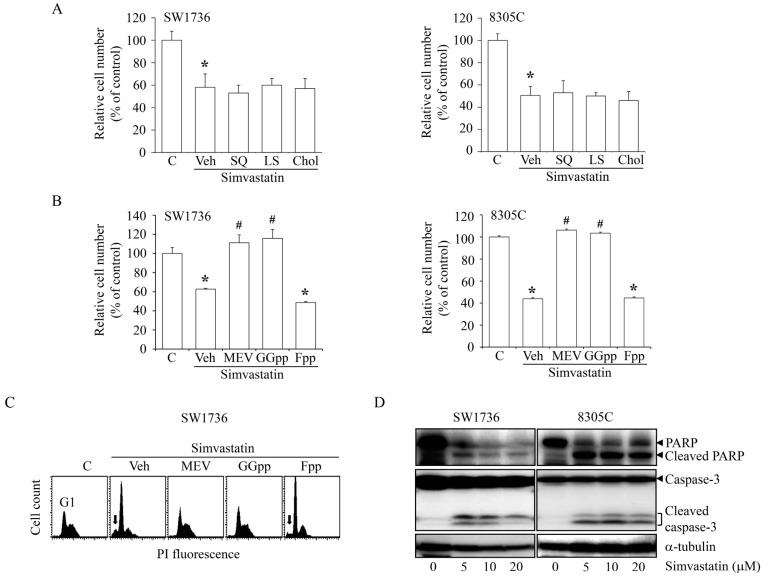
Effects of MEV-derived metabolites on the simvastatin-induced ATC cell proliferation inhibition. (**A**) Simvastatin (10 μM)-inhibited proliferation of SW1736 and 8305C cells was not affected by the add-in SQ (10 μM) or LS (10 μM) or Chol (10 μM). Cells were pre-incubated with SQ, LS or Chol for 30 min followed by simvastatin for additional 48 h. The simvastatin (10 μM) induced cell proliferation inhibition (**B**) and accumulation of cells at the G1-phase (**C**) of SW1736 and 8305C cells were abolished by MEV (50 μM) and GGpp (20 μM), but not Fpp (20 μM). Cells were pre-incubated with MEV, GGpp or Fpp for 30 min followed by simvastatin for additional 48 h. The relative cell number was estimated using MTT assays. Values represent the means ± SEM (*n* = 3). The DNA content was measured by PI staining. Arrows indicated sub-G1 cell population. (**D**) Simvastatin triggered apoptosis in SW1736 and 8305C cells. Cells were incubated with 0–20 μM simvastatin for 48 h, and then the protein expression levels of full-length and active form of caspase 3 and PARP were examined by immunoblotting analysis. * *p <* 0.05, different from corresponding control. ^#^ *p <* 0.05, different from simvastatin-incubated group. C, control; Veh: simvastatin-incubated group.

To study the involvement of the MEV pathway in simvastatin-induced cell proliferation inhibition, SW1736 and 8305C cells were co-incubated with simvastatin (10 μM) and MEV (50 μM) or MEV-derived metabolites. As illustrated in Figure 2A, the add-in squalene (SQ; 10 μM), lanosterol (LS; 10 μM) or cholesterol (Chol; 10 μM) had no significant effect on the simvastatin-inhibited cell proliferation, suggesting that the anti-proliferative effects of simvastatin might be due to the depletion of cholesterol and its intermediates. Both MEV and GGpp (20 μM), but not FPP (20 μM), can significantly rescue the simvastatin-induced anti-proliferation effect (Figure 2B). We further verified the effect of MEV and isoprenoids depletion on cell cycle progression. As demonstrated in Figure 2C, simvastatin treatment led to G1 arrest, and this effect was abrogated by co-treatment with MEV or GGpp, but not Fpp. These results suggest that the depletion of MEV or GGpp might contribute to the anti-proliferative activity of simvastatin, and implied that the geranylgeranylation pathway might play a role in the simvastatin-induced anti-proliferation in SW1736 and 8305C cells. Since simvastatin might increase the apoptotic cell populations at the sub-G1 phase (Figure 2C), we examined the protein expression levels of the apoptotic markers caspase 3 and poly (ADP-ribose) polymerase (PARP). As shown in Figure 2D, simvastatin at various concentrations (5–20 μM) induced the activation of caspase 3 and PARP, suggesting that simvastatin might cause not only cell proliferation inhibition but also apoptosis in ATC cells. 

### 2.3. Simvastatin Reduces the Acitivity of Rho GTPases

**Figure 3:**
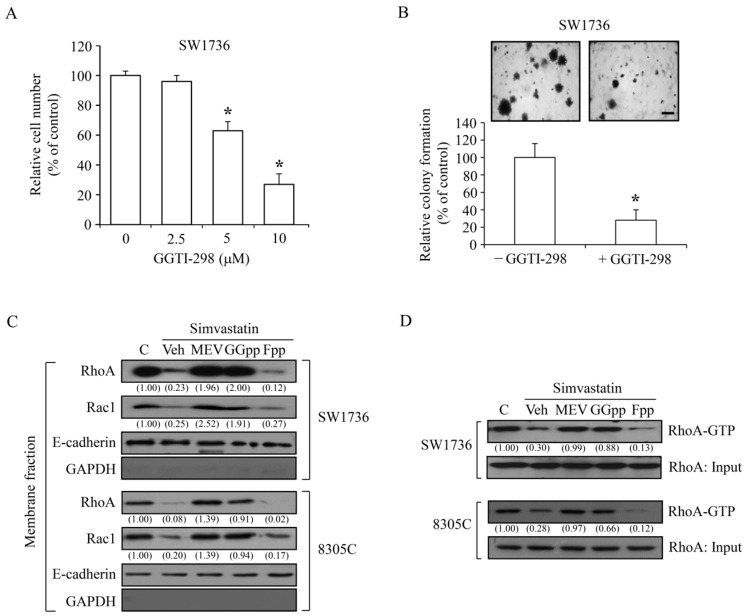
Effects of MEV, GGpp and Fpp on the simvastatin-induced inactivation of RhoA and Rac1 protein in ATC cells. (**A**) A specific geranylgeranyl transferase inhibitor GGTI-298 at concentrations of 5 μM and 10 μM significantly suppressed the cell proliferation in SW1736 cells. Cells were incubated with GGTI-298 for 48 h, and the relative cell number was accessed. Values represent the means ± SEM (*n* = 3). (**B**) GGTI-298 inhibited the cell growth of SW1736 cells in the semi-solid agar. Cells were incubated with GGTI-298 for 14 days, and then the formation of cell colonies were stained using crystal violet. Scale bar: 500 μm. Values represent the means ± SEM (*n* = 3). (**C**) Simvastatin (10 μM) decreased the amount of membrane translocation of RhoA and Rac1 protein, and this effect was abolished by pre-treatment with MEV (50 μM) or GGpp (20 μM), but not by Fpp (20 μM), in SW1736 and 8305C cells. Cells were incubated with simvastatin alone or in the presence of MEV, GGpp or Fpp for 48 h, and then the expression level of membrane-bound RhoA and Rac1 protein was analyzed. GADPH protein served as an indicator of non-membrane fractions. E-cadherin protein served as an indicator of membrane fractions. Values shown in parentheses represent the quantified results adjusted with corresponding E-cadherin protein level and expressed as ratio over control. (**D**) Simvastatin reduced the amount of RhoA-GTP active form, and this effect was prevented by MEV or GGpp, but not by Fpp, in SW1736 and 8305C cells. Cells were incubated with simvastatin alone or in the presence of MEV, GGpp or Fpp for 48 h, and then the RhoA-GTP active form was analyzed. Ten percent of total lysate was as loading input for RhoA protein. Values shown in parentheses represent the quantified results adjusted with corresponding RhoA-input protein level and expressed as ratio over control. * *p <* 0.05, different from corresponding control. C, control; Veh, simvastatin-incubated group.

The involvement of geranylgeranylation pathway was examined in an anchorage-dependent and anchorage-independent cell growth of SW1736 cells. As presented in Figure 3A, GGTI-298, a specific geranylgeranyl transferase inhibitor, significantly and concentration-dependently suppressed the anchorage-dependent proliferation. The cell colony formation was decreased by 80% under GGTI-298 (5 μM) treatment (Figure 3B). These findings suggest that molecules processed by post-translational prenylation might play a role in the simvastatin-induced inhibitory effect in the metastatic transition of ATC. Since the activation of Rho family of proteins is mainly through geranylgeranylated modification [25] and the results in Figure 2B,C showed that the add-in GGpp completely abolished the effect of simvastatin-induced anti-proliferation, we studied the effects of simvastatin and depletions of MEV, GGpp and Fpp on the activation of RhoA and Rac1 proteins. Immunoblotting analysis demonstrated that the amount of RhoA and Rac1 proteins in the membrane fractions of SW1736 and 8305C cells were significantly decreased by simvastatin; however, this effect was abolished by pre-treatment with MEV or GGpp, but not Fpp (Figure 3C). To further confirm that the RhoA activity was suppressed by simvastatin, a rhotekin-base pull-down assay was performed. As demonstrated in Figure 3D, the abundance of RhoA-GTP was decreased by simvastatin and this effect was abolished by pre-treatment with MEV or GGpp, but not Fpp. These results suggest that the RhoA/Rac1 signaling pathway might be involved in the simvastatin-induced anti-proliferation of SW1736 and 8305C cells. 

### 2.4. MEV and GGpp, but Not Fpp, Prevent the Simvastatin-Increased Expression of p21^cip^ and p27^kip^

**Figure 4:**
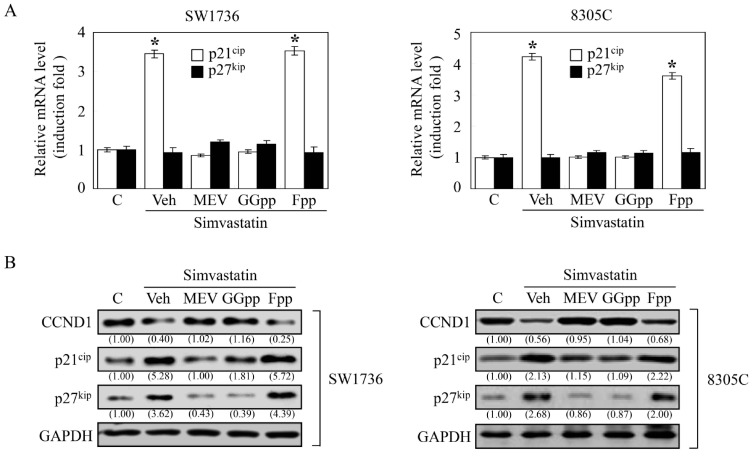

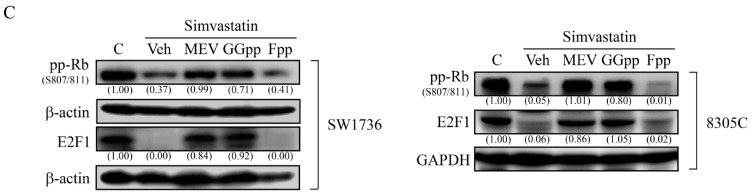
Effects of MEV, GGpp and Fpp on the simvastatin-increased p21^cip^ and p27^kip^ expression in ATC cells. (**A**) Simvastatin (10 μM) increased the mRNA expression level of *p21^cip^*. Values represent the means ± SEM (*n* = 3). * *p <* 0.05, different from corresponding control. Simvastatin increased the protein levels of p21^cip^ and p27^kip^ and decreased the protein levels of CCND1 (**B**), hyperphosphorylated-Rb and E2F1 (**C**). These effects were prevented by MEV (50 μM) or GGpp (20 μM), but not Fpp (20 μM). SW1736 and 8305C cells were incubated with simvastatin alone or in the presence of MEV, GGpp or Fpp for 48 h, and then the mRNA and protein levels of p21^cip^ and p27^kip^ were quantified and analyzed. GAPDH or β-actin proteins served as control for equivalent loading. Values shown in parentheses represent the quantified results adjusted with corresponding GAPDH or β-actin protein level and expressed as ratio over control. C, control; Veh, simvastatin-incubated group.

**Figure 5:**
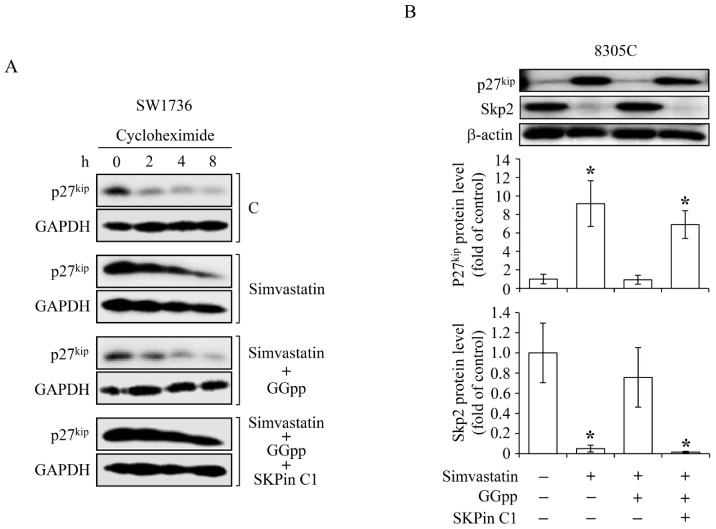
Effects of simvastatin on p27^kip^ protein stability in ATC cells. (**A**) Cycloheximide-induced degradation of p27^kip^ protein in SW1736 cells was retarded by simvastatin (10 μM), and the effect caused by simvastatin was revoked by GGpp (20 μM). The effect of add-in GGpp on p27^kip^ protein stability was eliminated by SKPin C1 (5 μM). Cells were incubated with DMSO or simvastatin for 40 h followed by cycloheximide incubation for additional 2–8 h. The p27^kip^ protein levels were then analyzed. (**B**) Simvastatin-augmented p27^kip^ protein level was eliminated by GGpp, and the effect caused by GGpp treatment was abolished by incubation of 8305C cells with SKPin C1. Cells were pre-incubated with GGpp and/or SKPin C1 for 30 min followed by simvastatin incubation for additional 48 h. The p27^kip^ and Skp2 protein level was then analyzed. Values represent the means ± SEM (*n* = 3). * *p <* 0.05, different from corresponding control. GAPDH or β-actin proteins served as controls for equivalent loading. C, control.

Both the over-expressed cyclins and under-expressed cyclin-dependent kinase inhibitors are crucial to the agitated cell growth and prone to oncogenesis [26]. It has been pathologically shown that cyclin D1 (CCND1) protein is overexpressed [27] and the protein expression levels of p21^cip^ [28,29] and p27^kip^ [30] were at low to rare levels in thyroid tumors, and these changes were attached to thyroid carcinogenesis. Accordingly, we investigated the effects of simvastatin on the expression of CCND1, p21^cip^ and p27^kip^. Analysis by quantitative reverse transcription PCR, simvastatin up-regulated the mRNA level of *p21^cip^*, but not *p27^kip^*, and this increase was prevented by pre-treatment with MEV or GGpp, but not Fpp (Figure 4A). Immunoblotting analysis showed a decreased protein level of CCDN1, and notably increased protein levels of p21^cip^ and p27^kip^ in the simvastatin-treated SW1736 and 8305C cells, and these effects were abolished by pre-treatment with MEV or GGpp, but not Fpp (Figure 4B). Since hyper-phosphorylation of Rb protein (pp-Rb), which unlatches E2F1 protein, plays an essential role in G1 checkpoint, we further estimated their protein expression levels. As presented in Figure 4C, simvastatin reduced the protein levels of pp-Rb and E2F1, and these effects were eliminated by MEV or GGpp, but not Fpp. These findings suggest that changes of CCND1, p21^cip^, p27^kip^, Rb, or E2F1 expression caused by simvastatin might potently lead to G1 arrest, and thereby inhibit cell proliferation in ATC cells. Unlike other tumor suppressor genes, p27^kip^ is infrequently mutated, and presents relatively lower levels in human cancers [31], suggesting that post-transcriptional regulatory mechanisms is involved in the disruption of p27^kip^ protein expression. Since the Skp2-dependent degradation of p27^kip^ protein obligates cancer cells to grow progressively [32,33], we checked the effect of GGpp depletion on Skp2-mediated p27^kip^ protein degradation. Protein stability analysis showed that simvastatin detained the p27^kip^ protein degradation, and this effect was abrogated by GGpp. However, the effect of add-in GGpp was retarded by the potent inhibitor SKPin C1 of Skp2-dependent p27^kip^ protein degradation (Figure 5A). As displayed in Figure 5B, simvastatin-induced prevention of p27^kip^ protein degradation and decreases of Skp2 protein level were eliminated by GGpp supplementation, and then was retrieved by co-incubation with GGpp and SKPin C1. These results suggest that simvastatin up-regulated p27^kip^ protein expression level via suppression of Skp2 action.

### 2.5. Inhibition of the EGF-Enhanced ATC Cell Migration by Simvastatin

**Figure 6:**
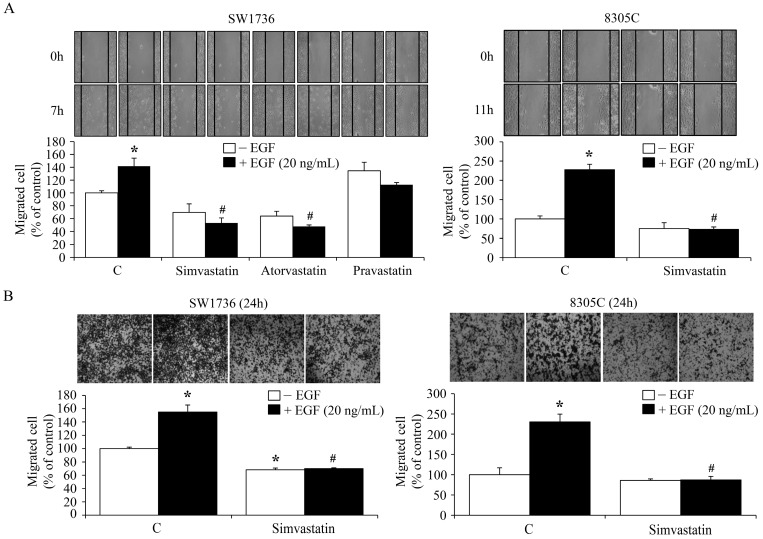
Effects of simvastatin on the EGF-enhanced ATC cell migration. Simvastatin (10 μM) diminished the migrated cell number of SW1736 and 8305C cells treated with or without EGF and evaluated using scratch assay (**A**) or Transwell assay (**B**). For horizontal cell migration, cells were pre-incubated with simvastatin for 1 h followed by a creation of migration gap and EGF administration in culture medium containing 2% FBS for additional 7 or 11 h. Cells in the migration gap were counted. For vertical cell migration, cells were pre-incubated with simvastatin for 4 h and then seeded into upper chamber of transwells containing 0% FBS. After 24 h the migrated cells attracted by the culture medium containing 5% FBS with or without EGF on the side of lower chamber of Transwell were counted. Values represent the means ± SEM (*n* = 3). * *p <* 0.05, different from corresponding control. ^#^ *p <* 0.05, different from EGF-incubated only group. C, control.

**Figure 7:**
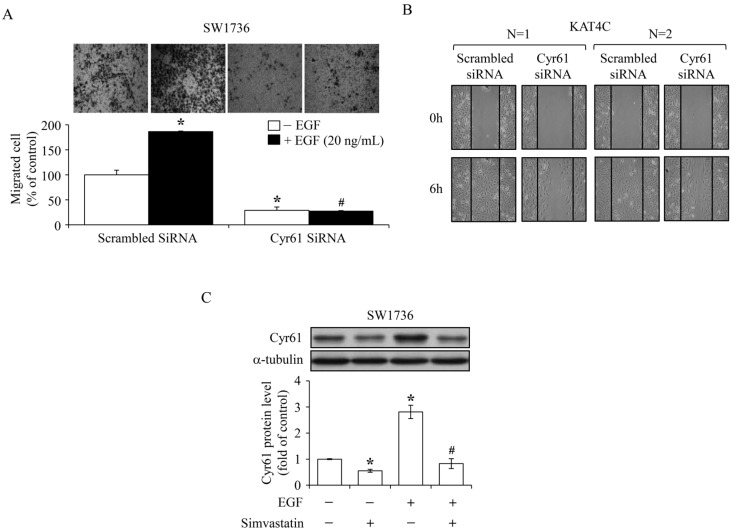
Role of Cyr61 in ATC cell migration. Introduction of *Cyr61* siRNA remarkably reduced the migrated cell number of SW1736 (**A**) and KAT4C (**B**) cells. In (**A**), after introduction of *Cyr61* siRNA (30 pmole) into SW1736 cells for 48 h, cells were seeded into upper chamber of Transwell containing 0% FBS. The medium in the bottom chamber contained 5% FBS. Cells were incubated in the Transwell for additional 24 h, and then the migrated cells attracted by the culture medium containing 5% FBS with or without EGF on the side of lower chamber of Transwell were counted. Values represent the means ± SEM (*n* = 3). * *p <* 0.05 and ^#^ *p <* 0.05, different from corresponding control. In (**B**), *Cyr61* siRNA (20 pmole) was introduced into KAT4C cells for 48 h followed by a creation of migration gap. Cells were incubated for additional 6 h, and then the cells in the migration gap were counted. Similar results were shown in two independent experiments. (**C**) Simvastatin (10 μM) prohibited the EGF-increased expression level of Cyr61 protein in SW1736 cells. Cells were pre-incubated with simvastatin for 4 h followed by EGF treatment for additional 1 h. The Cyr61 protein level was then analyzed. α-Tubulin served as control for equivalent loading. Values represent the means ± SEM (*n* = 3). * *p <* 0.05, different from corresponding control. ^#^ *p <* 0.05, different from EGF-incubated only group.

Previously, we demonstrated that Cyr61 protein played a central role in the EGF-enhanced cell migration of SW1736 cells [34]. In the present study, the effects of simvastatin on EGF-enhanced cell migration were further evaluated. To observe ATC cell migration, scratch (Figure 6A) and Transwell assays (Figure 6B) were conducted. As shown in Figure 6A,B, EGF prompted cell migration of SW1736 and 8305C cells, and these effects were inhibited by simvastatin. The migration scratch assay indicated that both simvastatin and atorvastatin could remarkably inhibit EGF-enhanced cell migration. However, pravastatin might have minor effect on EGF-enhanced SW1736 cell migration (Figure 6A). In agreement with our previous study, introduction of *Cyr61* siRNA into SW1736 cells significantly eliminated cell migration (Figure 7A). Moreover, knock-down of *Cyr61* in KAT4C cells also restrained cell migration (Figure 7B). Immunoblotting analysis demonstrated that simvastatin reduced both the endogenous level and the EGF-increased level of Cyr61 protein (Figure 7C). These findings suggest that decreased Cyr61 levels might be crucial for the simvastatin-inhibited cell migration induced by EGF.

## 3. Discussion

HMG-CoA reductase inhibitors have been used to minimize the incidence of cardiovascular events caused by hyperlipidemia [35]. In addition to the beneficial activity in plasma cholesterol reduction, the growth suppression effects of HMG-CoA reductase inhibitors, such as simvastatin, on various cancer cells have been indicated in several reports [36,37,38]. Nonetheless, the effects of simvastatin on ATC cells and its underlying regulatory mechanisms are still unclear. In the present study, our findings revealed that simvastatin reduced ATC cell proliferation through reduction of RhoA/Rac1 activity and up-regulation of p21^cip^ and p27^kip^. Furthermore, simvastatin abrogated EGF-enhanced ATC cell migration through the blockade of Cyr61 expression. To our knowledge, this is a novel demonstration that simvastatin reduced cell proliferation via increases of p21^cip^ and p27^kip^ expression and decreases of RhoA/Rac1 activation, and inhibited migration via and reduction of Cyr61 expression in human ATC cells. 

Simvastatin reduces MEV synthesis, leading to a depletion of its downstream isoprenoids. These MEV metabolic derivatives, such as Fpp and GGpp, play considerable roles in diverse cellular progressions, such as DNA replication and cell migration [39,40,41]. In the present study, pre-incubation with the downstream derivatives, Fpp, SQ and LS (those are for fatty acid synthesis), and its end product Chol did not rescue the effects of simvastatin on ATC cell proliferation inhibition (Figure 2A). Moreover, simvastatin-inhibited cell proliferation (Figure 2B) and G1 arrest (Figure 2C) in ATC cells were completely abolished by pre-treatment with MEV and GGpp, but not Fpp. These results suggest that depletion of MEV or GGpp might be mainly engaged in the anti-proliferative activity of simvastatin. Interestingly, the Fpp supplementation had no effect on ATC cell proliferation inhibition induced by simvastatin. One of the possible explanations is that Fpp itself is merely an intermediate for the synthesis of downstream products, but not as one of the effectors leading to ATC cell proliferation. This phenomenon deserves further investigation. As revealed in Figure 2C, simvastatin-increased cell populations at the sub-G1 phase in SW1736 cells was abolished by pre-treatment with MEV or GGpp, but not Fpp supplementation, suggesting that simvastatin might actuate the apoptotic process, and then contribute to the decreases of cell viability (Figure 1A). Normally, cells require a fine interaction with the matrices surrounding growth microenvironments, and then the productive cell growth is secured [42,43]. When cell–matrix bio-interactions are broken-down, cells will expire by arrest of cell cycle and admittance of apoptosis, precluding from dysplastic development [44]. However, tumor cells escape from these regulated processes and survive in an anchorage-independent condition by the liberation of guided proliferation and migration, leading to the colonization and metastatic growth [24,45]. As exhibited in Figure 1D, malignant ATC SW1736 cells presented an anchorage-independent growth in semi-soft media. Incubation with simvastatin significantly reduced this colony formation, suggesting an essential role for the MEV metabolic pathway in cell transformation and metastasis development.

Isoprenoid intermediates function as lipid moieties for the post-translational isoprenylation of a number of proteins, such as Ras and Rho family proteins. Hindrance of prenylation results in inactivation of Ras and Rho family proteins on the plasma membrane [46]. In the present study, we demonstrated that a selective inhibitor of geranylgeranylation GGTI 298 concentration-dependently decreased cell proliferation (Figure 3A) and dramatically reduced cell colony formation (Figure 3B) in ATC SW1736 cells. Additionally, GGpp, but not Fpp, supplementation significantly abolished the simvastatin-induced cell proliferation inhibition (Figure 2B) and G1 arrest (Figure 2C). These results suggest that activation of Rho family proteins by geranylgeranylation might play a major role in ATC cell growth. Simvastatin reduced the activation of small GTP-binding proteins on the plasma membrane. Supplementation with MEV and GGpp, but not Fpp, restored the simvastatin-inactivated RhoA and Rac1 proteins. These results suggest that simvastatin-induced ATC proliferation inhibition might be mediated by elimination of geranylgeranylation of RhoA/Rac1. Our previous study also showed that the lovastatin-induced proliferation inhibition in malignant ARO cells was irrelevant to the farnesylation of Ras protein [47]. We are going to investigate this issue to determine whether this effects is unique to all ATC cells induced by statin analogs or not in the future.

**Figure 8:**
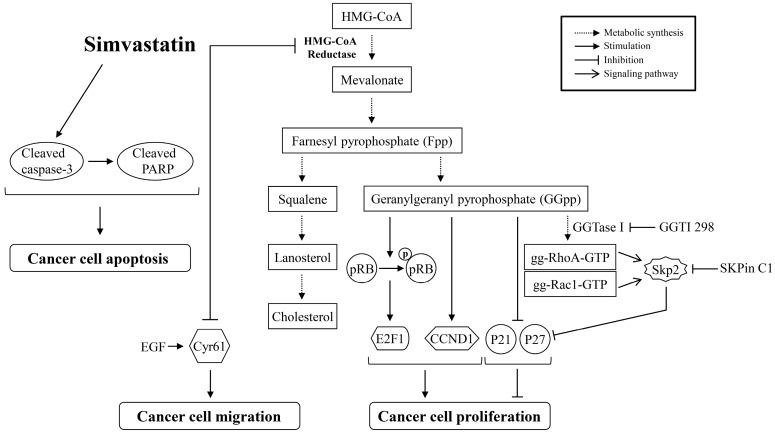
Simvastatin reduces cell proliferation and migration and triggers apoptosis in ATC cells. Incubation of ATC cells, SW1736 and 8305C, with simvastatin produces cell proliferation inhibition via inactivation of RhoA/Rac1 small GTPase protein, reduction of protein levels of CCND1, pp-Rb and E2F1, and increases of protein levels of p21^cip^ and p27^kip^. Simvastatin causes cell migration inhibition via repression of Cyr61 expression. In addition, simvastatin induces apoptosis via caspase 3 and PARP activation. gg-Rac1-GTP, geranylgeranylated-Rac1-GTP; gg-RhoA-GTP, geranylgeranylated-RhoA-GTP; pp-Rb, hyperphosphorylated-Rb.

During cell proliferation, the orchestrated regulation of guardians of G1-to-S-phase-transition is critical to accurate cell cycle progression. The Rb-E2F1 signaling is a potent inhibitory device. When Rb proteins are at hyperphosphorylated state, the physically latched E2F1 proteins will be disengaged from Rb-E2F1 complexes, and then the E2F1 transcription factor will enhance the expression of downstream genes coded for S phase entry [48]. Mutation of Rb gene has not been found in ATC cells, so the encoded Rb protein does not lose its ability to bind and inactivate E2F1 proteins [49,50]. As illustrated in Figure 4C, simvastatin decreased the expression levels of pp-Rb and E2F1 proteins. Supplementation of MEV or GGpp, but not Fpp, abolished simvastatin-decreased Rb protein hyperphosphorylation and E2F1 protein level, suggesting that depletion of MEV or GGpp might be involved in the positive regulation of Rb-E2F1 signaling. Both p21^cip^ and p27^kip^ are two of the key molecules to restrain the impaired DNA from being synthesized and propagated, thereby guarding the progression of the cell cycle [51]. However, immunohistochemical staining revealed that protein levels of p21^cip^ [28,29] and p27^kip^ [30] are at very low levels in ATC tissues, suggesting that p21^cip^ [28,29] and p27^kip^ may be associated with the uncontrolled cell cycle progression and tumorigenesis. As analyzed in Figure 4A, the simvastatin-increased *p21^cip^* mRNA level was abolished by pre-treatment with MEV or GGpp, but not Fpp. On the other hand, the *p27^kip^* mRNA level was not significantly changed under these incubations. The encoded p21^cip^ and p27^kip^ proteins were up-regulated by simvastatin, and these effects caused by simvastatin were eliminated by pre-treatment with MEV or GGpp, but not Fpp (Figure 4B). These results suggest that simvastatin up-regulated p21^cip^ expression at the transcriptional level and p27^kip^ expression at the post-translational level. The abundance of p27^kip^ protein is mainly controlled by the ubiquitin-mediated proteolysis [52,53]. It has been suggested that Skp2 is required for ubiquitin-mediated proteolysis of p27^kip^ protein [54], and Skp2-dependent proteolysis of p27^kip^ protein can promote cell cycle progression [32,33]. In the present study, we showed that the simvastatin-induced prevention of degradation and increased expression of p27^kip^ protein were reduced by GGpp supplementation, and these effects caused by simvastatin were eliminated by the Skp2-specific inhibitor SKPin C1. These findings suggest that simvastatin-induced up-regulation of p27^kip^ protein might be through the inhibition of a Skp2-dependent degradation pathway, which was a downstream event trigged by GGpp. In terms of Skp2-p27^kip^ regulation, there is a signaling linkage between Rho GTPase proteins and Skp2 expression. Transfection with constitutively active RhoA (RhoA14V) into human microvascular endothelial cells indicated that activation of RhoA decreased the levels of Skp2 and p27^kip^ proteins, resulting in G1 phase progression [55]. It has been shown that activation of Rac1 increases and stabilizes Skp2 protein, subsequently down-regulating the expression of p27^kip^ protein and finally stimulating proliferation of vascular smooth muscle cells [56]. Moreover, our previous results demonstrated that GGpp supplementation reduced the lovastatin-induced Skp2-mediated p27^kip^ degradation, and inactivation of RhoA substrate protein ROCK increased the p27^kip^ protein level in ARO cells [47]. These findings might support our hypothesis of RhoA/Rac1-Skp2-p27^kip^ signal axis in regulating the simvastatin-induced proliferation in ATC cells depicted in Figure 8. On the other hand, we did not further investigate the effect of Skp2 on p21^cip^ protein in the present study. However, the possible involvement of the Skp2-mediated degradation of p21^cip^ protein could not be excluded [57], and the transcriptional regulation of p21^cip^ expression by simvastatin still needs to be further elucidated [58].

We previously investigated the EGF-enhanced migration in ATC cells, and our findings revealed that an immediate-early gene *Cyr61* may play a vital role in this effect [34]. In the present study, we observed further the effect of simvastatin on the EGF-enhanced migration in ATC cells in vitro. As examined in horizontal and vertical migration activity of cells, simvastatin reduced the EGF-enhanced cell migration (Figure 6). Furthermore, simvastatin reduced not only basal but also the EGF-increased level of Cyr61 protein (Figure 7C). In agreement with our previous results, knock-down of *Cyr61* led to a significant loss of primary and EGF-stimulated activity of ATC cell migration (Figure 7A,B). These results suggest that Cyr61 might be a considerable target for simvastatin-induced cell migration inhibition. 

ATC cells do not express thyroid-specific markers (thyroid transcription factor-1, Paired box gene 8, iodide peroxidase, thyroglobulin and thyroid stimulating hormone receptor), and thereby contribute to an incapability of iodine transportation [59]. Re-differentiation of the advanced thyroid cancer cells could favor the restored sensitivity to radioiodide therapy [60]. Although we did not observe these markers for iodine uptake, it was shown that lovastatin at a concentration of 25 μM could provoke the cytomorphological differentiation and increases of the amount of secreted thyroglobulin in ARO cells [61]. Further investigation on the effects of simvastatin, which is the methylated form of lovastatin, on ATC differentiation may address this question. Since simvastatin targets the MEV pathway, which is essential for mitogenic signaling (e.g., Ras family proteins), we expect that simvastatin may be also effective in other types of thyroid cancer, including papillary thyroid cancer. Zeybek et al. investigated the effects of rosuvastatin on the papillary thyroid cancer cell line B-CPAP and the results showed that rosuvastatin could induce proliferation inhibition, G1 phase cell cycle arrest, ER stress and apoptosis, contributing to the death of B-CPAP cells [62]. Retinoic acid-based re-differentiation therapy was shown to be effective in recovering the function of radioiodide uptake in many types of thyroid cancer (e.g., papillary and follicular thyroid cancer) [63]. It would be interesting to examine whether there is an additive or synergistic effect using simvastatin in combination with retinoic acid on ATC. Our previous results indicated that lovastatin could induce apoptosis at 50 μM [64], reduce the EFG-induced invasiveness at 20 μM [65], and inhibit proliferation at 20 μM in ARO cells [47]. In the present study, we showed that 10 μM of simvastatin could induce proliferation inhibition and apoptosis, reduce relative colony formation, and suppress the EGF-enhanced migration in ATC SW1736 cells. Treatment with 10 μM of simvastatin also inhibited cell proliferation and migration in ATC 8305C cells. These findings suggest that simvastatin might have a strong potency for thyroid cancer treatment.

## 4. Materials and Methods

### 4.1. Chemicals, Reagents and Antibodies

Simvastatin was purchased from Merck (Taipei, Taiwan). EGF was purchased from PeproTech (Rocky Hill, NJ, USA). Bovine serum albumin (BSA) was purchased from BioShop Canada (Burlington, ON, Canada). SKPin C1 was purchased from Tocris Bioscience (Avonmouth, Bristol, UK). 3-(4,5-Dimethylthiazol-2-yl)-2,5-diphenyltetrazolium bromide (MTT), GGTI-298, MEV, GGpp, Fpp, SQ, LS and Chol were purchased from Sigma-Aldrich (St. Louis, MO, USA). Unless otherwise indicated, all other chemicals were also purchased from Sigma-Aldrich. RPMI and MEM culture medium, kanamycin sulfate, fetal bovine serum (FBS), TRIzol reagent and SuperScript III Reverse Transcriptase system were purchased from Thermo Fisher Scientific (Waltham, MA, USA). Non-liposomal transfection reagent II (T-Pro NTR II) was purchased from Ji-Feng Biotechnology (Taipei, Taiwan). Antibody against Cyr61 was purchased from GeneTex (Hsinchu, Taiwan). Antibodies against α-tubulin and β-actin were purchased from Jackson Immuno Research Laboratories (West Grove, PA, USA). Cyr61 siRNA (cat. sc-39331), scrambled SiRNA (cat. sc-37007) and antibodies against CCND1, E2F1, E-cadherin, GAPDH, p21^cip^, p27^kip^, Rac1 and pp-Rb (S807/811) were purchased from Santa Cruz Biotechnology (Santa Cruz, CA, USA). Antibody against RhoA was purchased from Cytoskeleton (Denver, CO, USA). Antibody against caspase 3 and PARP were purchased from Cell Signaling Technology (Danvers, MA, USA).

### 4.2. Cell Lines and Culture Conditions

Human ATC SW1736 (a gift from Dr. N. E. Heldin, University of Uppsala, Uppsala, Sweden), 8305C (a gift from Dr. P. Vigneri, University of Catania, Catania, Italy) and KAT4C (a gift from Dr. K. B. Ain, University of Kentucky Medical Center, Lexington, KY, USA) cells were grown in RPMI medium containing 5% FBS and 1% kanamycin at 37 °C in a humidified culture incubator containing 5% CO_2_. WI-38 human lung fibroblast cells were grown in MEM medium containing 10% FBS and 1% penicillin-streptomycin at 37 °C in a humidified culture incubator containing 5% CO_2_. The concentrations of chemical compounds used for cell treatments were as follows: simvastatin (10 μM); EGF (20 ng/mL); MEV (50 μM); GGpp (20 μM); Fpp (20 μM); SQ (10 μM); LS (10 μM); Chol (10 μM); GGTI-298 (5 μM); SKPin C1 (5 μM). 

### 4.3. Cell Viability and Proliferation Assay

SW1736 and 8302C cells (1 × 10^6^ cells/mL) were seeded in 24-well plates for 24 h followed by incubation with simvastatin or GGTI-298 at various concentrations for additional 48 h. In other experiments, cells were co-incubated with simvastatin and MEV, GGpp, Fpp, SQ, LS, or Chol separately for 48 h. At the end of incubation, a 500 μL volume of MTT reagent (1 mg/mL) was added into each culture well and incubated for 20 min. The medium was then removed, and the intracellular purple formazan dissolved in DMSO was measured at 570 nm in a microtiter plate reader (BioTek Instruments, Winooski, VT, USA). Absorbance at 430 nm served as a reference wavelength.

### 4.4. Anchorage-Independent Growth Assay

SW1736 cells (2 × 10^4^/well) were mixed with 0.36% agarose (BD Difco Agar Noble; Sparks, MD, USA) and plated on the top of 0.72% agarose gel in RPMI 1640 medium containing 5% FBS and 1% kanamycin in 6-well plates. Cells were incubated with GGTI-298, simvastatin at various concentrations, or a combination of simvastatin and MEV or GGpp or Fpp for 14 days. The cover medium was refreshed every two days. Colonies were stained by 0.25% (*w*/*v*) crystal violet. Dark blue cell colonies were counted and presented as an index of anchorage-independent growth.

### 4.5. Cell Cycle Analysis

The population of SW1736 cells at each phase of cell cycle was measured by the discriminative DNA content stained with propidium iodide (PI). Cells were fixed with chilled 70% ethanol and kept in a −20 °C container. After 12 h, cells were washed with cold phosphate-buffered saline (PBS), and then the RNA were removed by incubation of the cells with 2 mg/mL RNase A at 37 °C for 30 min. To stain cellular DNA, cells were incubated with PI (50 μL/mL solution) at room temperature for 1 h in the dark. DNA content was recorded by FACSCalibur flow cytometer (Becton Dickinson, San Jose, CA, USA) and then analyzed by CellQuest software v3.3 (Becton Dickinson, Franklin Lakes, NJ, USA).

### 4.6. Extraction of Membrane-Bound Proteins

At the end of incubation with chemical compounds, cells were lysed in a hypotonic buffer (20 mM Tris-HCl, pH 7.5; 5 mM EGTA; 2 mM EDTA; 1 mM NaVO_3_; 1 mM dithiothreitol; 10 mM NaF; 10 mM Na_2_H_2_P_2_O_7_) containing protease inhibitor cocktail and kept in a −80 °C container. Two hours later, cell lysates were disrupted by a 3/10 mL BD^TM^ insulin syringe with a 30-gauge needle and the sediments were collected after centrifugation at 13,500× *g* for 15 min at 4 °C. Sediments containing membrane-bound proteins were extracted by a HEPES buffer (50 mM HEPES, pH 7.0; 250 mM NaCl; 2.5 mM EDTA; 1% Nonidet P-40; 5% glycerol; 1 mM NaVO_3_; 10 mM NaF; 10 mM Na_2_H_2_P_2_O_7_) containing protease inhibitor cocktail. The supernatant was then collected as the membrane fraction after centrifugation at 13,500× *g* for 40 min at 4 °C.

### 4.7. Measurement of RhoA Activity

The active form of RhoA protein, RhoA-GTP, was detected using Cytoskeleton GST-Rhotekin-RBD pull-down kit (Denver, CO, USA), and the assay was conducted according to manufacturer instructions.

### 4.8. Immunoblotting Analysis

Total cell lysates were extracted by Upstate Mg^2+^ lysis buffer (Merck Millipore, Darmstadt, Germany), and then dissolved in 7~12% gels of sodium-dodecyl-sulfate-polyacrylamide-gel-electrophoresis (SDS-PAGE). The proteins were then transferred onto Amersham Hybond-P polyvinylidene difluoride (PVDF) membranes (GE Healthcare Life Science, Piscataway, NJ, USA). Indicated proteins were hybridized with primary antibodies in 3% BSA solution, washed with tris-buffered saline containing 0.1% tween 20, and then probed with horseradish-peroxidase (HRP)-labeled secondary antibodies. The emitted signal of target proteins was developed by chemiluminescence reagent plus (PerkinElmer Life Sciences, Boston, MA, USA) and identified by BioSpectrum Imaging System (UVP, LLC Upland, CA, USA).

### 4.9. Measurement of mRNA Levels of p21^cip^ and p27^kip^

Trizol reagent isolation of total cellular RNA was performed according to manufacturer instructions. Two micrograms of total RNA were subjected to first strand complementary DNA synthesis using SuperScript III Reverse Transcriptase system. The SYBR Green I-bound amplicons of target genes were detected and quantified by LightCycler Nano System (Roche Molecular Biochemicals, Mannheim, Germany). Primers used in real-time polymerase chain reactions were as follows-*p21^cip^* (RTPrimerDB ID 7843) 5′-CTGTCTTGTACCCTTGTGCC-3′ and 5′-GGTAGAAATCTGTCATGCTGG-3′; *p27^kip^* 5′-TGCAACCGACGATTCTTCTACTCAA-3′ and 5′-CAAGCAGTGATGTATCTGATAAACAAGGA-3′. 

### 4.10. Introduction of Cyr61 Small Interfering RNA (siRNA)

The mixtures of *Cyr61* siRNA or scrambled siRNA with T-Pro NTR II reagent were drop-wisely added into cultured cells, and further incubated for 48 h. Cells were then subjected to indicated experiments. The transfection procedures were performed according to manufacturer instructions.

### 4.11. Scratch Migration Assay

Confluent cells cultured in 12-well plates were subjected to migration-gap-creation by a steriled 200 μL yellow tip. To make a width-limited scratch in the middle of each culture well, the pipette tip was kept in an angle of around 45 degrees during creation of a migration gap. Scraped wells were then washed with PBS two times followed by the indicated incubations. The migrated cells were photographed and counted using Olympus IX70 microscope system (Tokyo, Japan). 

### 4.12. Transwell Migration Assay

The Costar Transwell plate with 8 μm pore size of polycarbonate membrane (Tewksbury, MA, USA) was used for the observation of vertical cell migration. Cells (3.5 × 10^4^/well) were seeded onto the upper chamber of Transwell containing serum-free RPMI culture medium. Cell migration was driven by 5% FBS with or without EGF in lower chamber of Transwell. After 24 h, the migrated cells on the other site of membranes were fixed with 4% paraformaldehyde, stained with 0.1% crystal violet, and counted.

### 4.13. Statistical Analysis

All data were expressed as the mean ± standard error of the mean (SEM). Experimental data were analyzed using Student’s two-tailed *t*-test. As a *p* value < 0.05, significance was statistically accepted.

## 5. Conclusions

The results from the present study indicate that the decreasing GGpp level is a key mechanism accounting for the anti-cancer effects of simvastatin in ATC. Simvastatin inhibited cell proliferation and colony formation and induced cell cycle arrest, while adding the exogenous GGpp rescued all these effects. Overcoming the inhibitory effects of simvastatin, GGpp also rescued the active proportion of geranylgeranylation-dependent RhoA and Rac1 proteins, reversing the protein profiles associated with cell cycle arrest including p21^cip^, p27^kip^, CCND1, pp-Rb and E2F1. Simvastatin could reduce metastasis-related activity by inhibiting Cyr61, a factor involved in the EGF-enhanced migration. Moreover, simvastatin might also trigger apoptosis. Based on our results, a molecular mechanism underlying simvastatin-suppressed ATC through inhibition of geranylgeranylation-dependent functions and Cyr61 expression, and apoptosis induction was proposed as depicted in Figure 8. The findings from these results suggest that simvastatin might potentially be a potent drug for ATC treatment. Since isoprenylation is important for the function of many small GTPases (e.g., Ras, RhoA), which are crucial for the cell proliferation and survival functions, it is very likely that their perturbed function can directly or indirectly affect the energy status and associated metabolic processes. It is noteworthy that Mullen et al. suggested the potentiation of IGF-1/AKT signaling being crucial to maintain the normal mitochondrial function and be free from simvastatin-induced damage in mitochondrial respiratory chain. In the study, they found that the increased phosphorylation level of AKT is the determinant to reduce mitochondrial susceptibility to simvastatin impairment [66]. Indeed, the mutated gene profiles in thyroid cancer will result in the overaction of MAPK and PI3K-AKT signaling pathway, subsequently inhibiting the critical thyroid iodine-uptaking process in thyrocytes, and thereby repress the synthesis of thyroid hormones [67]. Furthermore, blockade of the PI3K-AKT signaling could decrease the Rb-E2F1 activity and increase the protein levels of p21^cip^ and p27^kip^, contributing to the G1-phase cell cycle arrest [68]. We consider roles of upstream regulators that would be better addressed in understanding the involvement of particular signaling pathway that contribute to the anti-cancer effects of simvastatin in ATC. We are planning experiments in this direction of research in the future. 

## Abbreviation

DMSO	Dimethyl sulfoxide
